# Educational Technologies for Teaching Social Skills to Individuals With Schizophrenia: Scoping Review

**DOI:** 10.1177/15394492221108389

**Published:** 2022-07-26

**Authors:** Nicole Surdyka, Amy Clark, Andrea Duncan

**Affiliations:** 1 University of Toronto, Ontario, Canada

**Keywords:** educational technology, social participation, schizophrenia

## Abstract

Schizophrenia interventions incorporate improving quality of life and social functioning. Educational technologies are a potential treatment method for social skills development among individuals with schizophrenia. The objective of the study is to provide an overview of the characteristics and range of approaches of educational technologies in the context of social skills for individuals with schizophrenia. A scoping review methodological framework was applied. Search strategy was conducted on Ovid MEDLINE® and CINAHL Plus. Data were synthesized using a charting form for a logical, descriptive summary of results. The search yielded 771 results and 23 included studies that met eligibility criteria. The data showed persons with schizophrenia respond well to educational technologies to address illness self-management. Using technology in conjunction with traditional evidence-based interventions demonstrates promising results to improve social skills functioning. Occupational therapists can use educational technologies to decrease the gap in health care services and improve social support for individuals with schizophrenia.

## Introduction

Schizophrenia (SZ) is a serious mental illness with common symptoms that affect social functioning (Torous & Keshavan, 2016). Individuals with SZ may have altered perspectives due to symptoms affecting social functioning and the major determinant of functional impairment lies in social skills (SS) deficits (Torous & Keshavan, 2016). SS consists of verbal and non-verbal behaviors that allow individuals to communicate, adapt to social situations, and maintain emotional awareness (Reich, 2017). Deficits in SS and social functioning, including difficulties communicating with others, maintaining relationships, and functioning in the community can be hindered due to symptoms of SZ (Couture et al., 2006).

In considering these social needs, occupational therapy (OT) treatments aim to increase independence in activities of daily living by addressing SS to create and maintain social relationships among individuals with SZ and extend beyond symptom reduction and focus on improving quality of life (QoL) and social interactions (Bridges et al., 2013). Our psychosocial interventions include psychological, cognitive, social, and cultural aspects pertaining to social experiences that influence behaviors and engagement in occupations (American Occupational Therapy Association [AOTA], 2002).

Despite evidence on the efficacy of psychosocial interventions, individuals with SZ experience long-standing SS deficits and often lack access to evidence-based psychosocial interventions, particularly those targeting social functioning and QoL (Fulford et al., 2021). Access is limited due to cost, duration, and intensity of existing interventions (Fulford et al., 2021). This contributes to a treatment gap for SZ, adversely affecting long term SS, illness self-management of symptoms and motivation for functional-based care (Westermann et al., 2020). To address this, a promising treatment method to teach SS virtually to individuals with SZ is through the use of educational technology (ET).

ETs include software and media that deliver audio, images, and video to facilitate learning through structured and personalized, technological programs and resources (Kurt, 2015). Common ETs include internet, computer, virtual reality (VR), video games, and applications (Colder Carras et al., 2014). Implementation of ETs may offer an efficient and emerging method to teach SS and decrease the access gap to evidence-based psychosocial mental health care for people with SZ (Thompson et al., 2018). Previous studies have demonstrated feasibility, efficacy, and satisfaction with existing smartphone interventions (Ben-Zeev et al., 2014) and VR as an effective SS training program for people with SZ (Adery et al., 2018). There are currently no studies that systematically map the type of technology, features, and application of ETs used to address SS for SZ.

## Method

A scoping review methodology was used to identify the types of technology, features, and characteristics, application, and results of evaluations of ETs used to teach SS to individuals diagnosed with SZ (Arksey & O’Malley, 2005).

The three concepts that the search terms were derived from include mental illness, SS, and technology. After an iterative process that involved generating several search terms from the literature, navigating Yale MeSH Analyzer and consultation with a librarian, a search strategy was created. Search terms were inputted into the databases Ovid MEDLINE® and CINAHL Plus. The search terms were tested (Supplemental Appendix A), inclusion and exclusion criteria were refined (Supplemental Appendix B) and the list of articles was created in December 2020.

Studies were imported into Covidence to support unbiased screening and title and abstracts were first independently screened by the primary researchers. The primary researchers then independently conducted full-text reviews of the studies that met study criteria. Conflicts were resolved during weekly meetings and a third reviewer made the final decision.

A data charting form was created for a logical and descriptive summary of the results, which included intervention, type of technology, study population, outcome measures, and results of the studies. Throughout the data extraction process, the research team met regularly to develop consensus on the core themes present within the literature, which were discussed until consensus was reached.

## Results

**Figure 1.**
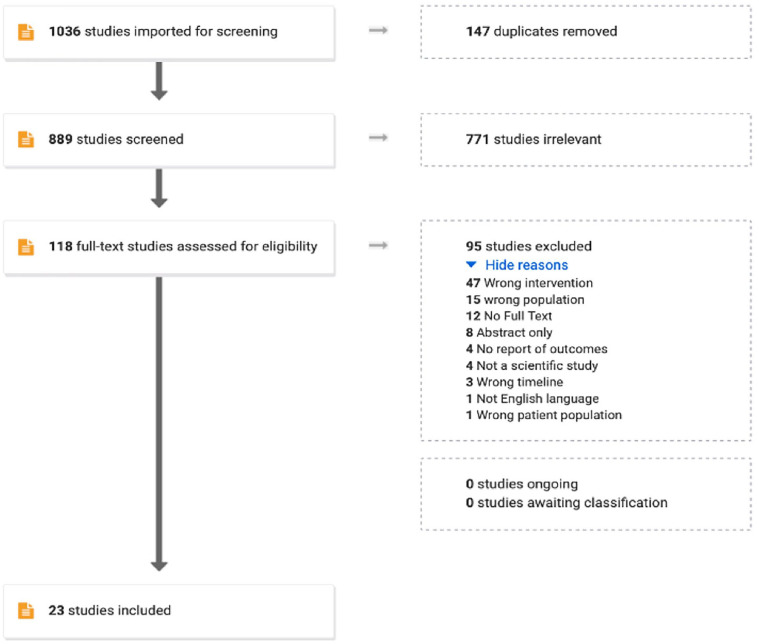
PRISMA flow chart. *Note.* Demonstrates the screening and study selection process using the inclusion and exclusion criteria.

The initial search resulted in 1,036 studies that were imported for screening. After the screening and selection process, 23 articles satisfied criteria and were included in the study (Figure 1). All the articles were published between 2007 and 2020 and 69% (*n* = 16) published on or after 2017. The types of studies included were one meta-analysis, seven randomized controlled trials (RCTs), and 14 pilot studies. The duration of the studies ranged from 1.5 hours to 24 weeks (Table 1).

**Table 1.** Study Characteristics.

Title	Authors/year published	Intervention and technology type	Study populations	Aims	Methodology	Outcome measures	Results
The efficacy of computerized cognitive drill and practice training for patients with a schizophrenia-spectrum disorder: a meta-analysis	Prikken et al. (2019)	Meta-analysis using Computerized Cognitive Drill and Practice Training	*N* = 1,262 Dx = SZ or SSD	To assess the efficacy of computerized drill and practice cognitive remediation to evaluate improvements on social cognition, psychotic and depressive symptoms, and functional outcomes.	A systematic search was carried out using PubMed, Embase, Cochrane Database of Systematic Reviews, and PsycINFO.	Outcomes were cognitive functioning, psychotic symptoms, depressive symptoms, and functional outcomes.	No strong evidence for improvements in general cognition, social cognition, and functional outcomes.
VR-based conversation training program for patients with schizophrenia: a preliminary clinical trial	Ku et al. (2007)	Preliminary Clinical Trial using iVR	*N* = 10 Dx = SZ	Tested VE to train people with SZ to develop conversational skills in specific situations to complement conventional SST or role-playing techniques.	Participants were trained in a VE. The scenario had four steps and was designed to cover conversations of those involved in normal conversation. Each step contained 10 tasks. After completing the program, they were asked to complete several questionnaires on social presence, and usability.	PANSS and SQ	A VR-based conversational training system could be used for conventional SST. Subjects feel presence in the VE and humanlike avatars that can provide emotional stimuli.
A virtual reality-integrated program for improving social skills in patients with schizophrenia: a pilot study	Rus-Calafell et al. (2014)	Pilot study using nVR	*N* = 12 DX = SZ or SSD	To explore the effectiveness and utility of a VR-integration program as an adjunct technique to deliver an individual cognitive-behavioral SST intervention (vs. group-based SST).	16 one-to-one 1-hr sessions twice a week. The more basic skills were consolidated (e.g., facial emotion recognition and social processing), and then addressed more complex skills (such as maintaining conversations). Complemented with the Soskitrain program, which is a VR program.	PANSS, AI, SSIT, SADS, SSFS, and Soskitrain	High acceptance of the VR system. Participants demonstrated a significant decrease in psychopathology and social avoidance and an improvement in SS which were maintained at follow-up. The improvement of conversation skills and the reduction of social anxiety demonstrate an increase in the confidence when conversing with others. The intervention may be effective for improving social dysfunction.
A virtual reality application in role-plays of social skills training for schizophrenia: a randomized, controlled trial	Park et al. (2011)	RCT using iVR	*N* = 91 Dx = SZ	To explore the advantages of the use of VR in social rehabilitation for patients with SZ.	SST using SST-VR and SST-TR was administered to groups of four to five members. Each session included a therapist to model behavior, followed by participant’s role-playing, and then receiving feedback. Participants then engaged in role-play of the same scene. Every session included three role-plays with different scenes.	SBS, RAS, RCS, and SPSI-R	The SST-VR group demonstrated greater interest in SST and generalization of the skills than the SST-TR group. After SST, the SST-VR group improved more in conversational skills and assertiveness than the SST-TR group, but less in nonverbal skills. It is considered a useful supplement to traditional SST.
A novel, online social cognitive training program for young adults with schizophrenia: a pilot study	Nahum et al. (2014)	Pilot study using computer-based training program	*N* = 34Dx = SZ or SSD	To test the feasibility of use, and efficacy, of a neuroplasticity-based online training program called SocialVille.	Participated in the SocialVille program at home or in clinic. The intervention included 24 hr of SocialVille game play (1–2 hr per day, 2–5 days per week over 6–12 weeks). Each SocialVille session consisted of 6 exercise blocks, each taking about 10 min to complete.	BIS/BAS, TEPS, and Post-Training Assessment Battery.	High satisfaction, enjoyment, and ease of use as rated by participants and demonstrated improvements on measures of social cognition, social functioning, and motivation. Demonstrated feasibility and resulted in within-subject gains in social functioning. These findings suggest that an online training approach is feasible.
Habits and attitudes of video gaming and information technology use in people with schizophrenia: cross-sectional survey	Choi et al. (2020)	Cross-sectional survey and pilot study using web-based video game	*N* = 110 Dx = SZ or SSD	To explore the habits and attitudes regarding video gaming and information technology use and their associated factors.	79-item self-report cross-sectional survey was distributed to participants to address factors associated with their gaming attitudes. Two questionnaires were distributed to participants with an interval of 2 weeks.	GAMES	Information technology and video gaming could be used to cope with stress and provide SST to people with SZ. Autonomy was the most common motive for video gaming among the participants, with “social playing,” as a motivating factor for social interaction and autonomy. Social bonds in virtual communities could help the development of interpersonal skills in the real world. This shows the potential of utilizing video games to facilitate interpersonal skills.
One-year randomized controlled trial and follow-up of integrated neurocognitive therapy for schizophrenia outpatients	Mueller et al. (2015)	RCT using computer-based training program	*N* = 156 Dx = SZ or SSD	To evaluate the efficacy of INT after therapy and after follow-up compared with TAU.	INT consisted of 30 sessions occurring bi-weekly for 90 min. All neuro- and social-cognitive targets were divided into four therapy modules and increased in complexity throughout the experiment. Homework was assigned to promote transfer of the cognitive skills into patients’ daily lives to maintain treatment effects.	PANSS, PFA, Emorec, SCST-R, and AIHQ	INT resulted in significantly larger effects in global social cognition, motion perception and social schema compared TAU at post-therapy and at 9 months. Integrated interventions on neurocognition and social cognition may also improve functional outcome.
Efficacy of cognitive rehabilitation using computer software with individuals living with schizophrenia: a randomized controlled trial in Japan	Iwata et al. (2017)	RCT using computer-based training program	*N* = 60 Dx = SZ	To determine whether a CR program is effective in improving both cognitive and social functions in SZ through learning-based psychiatric rehabilitation.	Included 24 cognitive training sessions using computer software (CogPack; Japanese version) administered twice a week (and an additional weekly group session over 12 weeks) with each session lasting 45 to 60 min. In addition to the intervention, almost all of the participants were attending day treatment services, and other psychosocial treatment were provided for 6 hr a day, 5 days per week.	PANSS, BAC-J, and LASMI	Providing CR in addition to usual treatment involving psychiatric rehabilitation approaches such as SST was associated with greater improvement in social functioning in people with SZ, compared with psychiatric rehabilitation services alone. Changes from pre-treatment to post-treatment in interpersonal relationships were significantly correlated.
Mobile enhancement of motivation in schizophrenia: a pilot randomized controlled trial of a personalized text message intervention for motivation deficits	Luther et al. (2020)	Pilot RCT using mHealth	*N* = 56 Dx = SSD	To test the feasibility and preliminary effectiveness of MEMS, a mobile text-messaging intervention, that leverages mobile technology to target motivation deficits with text messages.	Participants in the MEMS group received personalized, interactive text messages on cellphones each weekday through TextIt’s web-based text-messaging service. Three text message sets were sent plus a one-to-one goal setting session of approximately 45 min.	PANSS, GCT, QLS, MAP-SR, Usability, Satisfaction, and Ease of Use Questionnaire, and Strauss-Carpenter Level of Function scale	Retention, engagement, and satisfaction in MEMS were high. Participants had significantly greater improvements in interviewer-rated motivation and attained significantly more recovery-oriented goals at 8 weeks. MEMS indicated to be feasible as a brief, low-intensity mobile intervention that could improve aspects of motivation. Social interaction and accountability provided through the text messages may have led to the improvements.
A web-based game for teaching facial expressions to schizophrenic patients	Gülkesen et al. (2017)	Research study using web-based game	*N* = 32 Dx = SZ	To examine if a computer games website designed for teaching facial expressions could improve facial expression recognition skills of patients with schizophrenia.	The ILFE software, composed of 8 serious games for 1 month, 2 times per week for a 60-min period each. Six basic face expression images and brief explanations about these expressions were present in the training module. The module was developed for patients who wanted to practice before playing the games or between the games.	SANS, SAPS, BPRS, SDLT, WCST, and Porteus Labyrinths	Computer games may be used for the purpose of educating individuals with schizophrenia who have difficulty in recognizing facial expressions.
An ecological momentary intervention incorporating personalized feedback to improve symptoms and social functioning in SSDs	Hanssen et al. (2020)	RCT using mHealth	*N* = 50 Dx = SSD	To examine the feasibility and effectiveness of an interactive smartphone application that aimed to improve daily-life social functioning with ESM derived of personalized feedback prompts in response to participants’ answers on the ESM questionnaires.	Intervention included 6 short ESM questionnaires daily when prompted. Participants were asked to complete a personal-items-checklist regarding their favorite activities, coping mechanisms and social contacts. The SMARTapp was identical for both groups, except that one group received personalized interactive ESM feedback in the form of two tailored prompts a day. After 3 weeks participants attended the second session during which they completed the post-measures.	PANSS, CAPE, SFS, and WAIS	Receiving personalized feedback was associated with a reduction in feelings of loneliness. Smartphone-based applications with personalized feedback offer opportunities for feasible, simple, and accessible interventions for individuals with SSD.
Immersive virtual reality applications in schizophrenia spectrum therapy: a systematic review	Bisso et al. (2020)	Systematic review using iVR	*N* = N/A Dx = SZ	To investigate the use of iVR in the treatment of psychotic symptoms including delusions and paranoia, auditory verbal hallucinations, cognitive deficits, and SS.	Conducted according to PRISMA guidelines for Systematic Reviews. Searched the databases Web of Science, EMBASE, PsycINFO and CINHAL.	N/A	One RCT used iVR for increasing SS. The effectiveness of VR SST was compared against TR-SST was compared and VR SST demonstrated greater improvements in the conversational skills, assertiveness and motivation to treatment than TR-SST. VR SST was less effective in improving vocal and non-verbal skills than TR-SST. The evidence suggested its effectiveness to treat psychotic symptoms including SS.
Mobile-assisted cognitive behavioral therapy for negative symptoms: open single-arm trial with schizophrenia patients	Granholm et al. (2020)	Single-arm, open-trial, pre–post evaluation study using mHealth	*N* = 31 Dx = SZ or SSD	To investigate if mCBTn would lead to a significant reduction in severity of defeatist attitudes through an increase in engagement.	Involved weekly, 90-min, group therapy sessions with 2 therapists. Modules provided the core content for cognitive behavioral therapy for negative symptoms (mCBTn) through the CBT2go application. Mobile interactions occurred in the morning, with reminder prompts (for engagement) at midday and evening.	DPAS, ABS, CAINS-MAP, CDS, PANSS, A-QLS, and SFS	Significant improvement on the SFS total score from the beginning to 12 weeks but was not seen at 18 and 24 weeks. Large improvements in defeatist attitudes and negative symptoms which indicated feasibility and engagement. Recovery for goals could be motivating and engaging factors of the app. Participants responded to prompts to make an action plan about 1.5 times per week which is greater homework adherence than expected with the CBT group therapy alone.
Feasibility, acceptability, and preliminary efficacy of a smartphone intervention for schizophrenia	Ben-Zeev et al. (2014)	Quantitative study using mHealth	*N* = 30 Dx = SZ or SSD	To investigate the acceptability, usability, engaging, and helpfulness of the FOCUS mHealth application.	Participants chose three treatment targets (symptom management, mood regulation, medication adherence, social functioning, and improved sleep) out of five to work on by receiving daily prompts and content. Participants completed an assessment 3 times a day, about one of the three treatment targets. Participants were asked to respond to generated assessments and in response to the content of participant responses, the FOCUS system deployed tailored interventions. Follow-up was 1 month post trial.	PANSS, BDI-2, ISI, BMQ, and SQ	Participant engagement was high, participants used the application an average of 5.2 times a day with 52% initiated by the participants and 38% was in response to automated prompts. Approximately 90% of participants rated the intervention as highly acceptable and usable. Results indicate after using FOCUS for 1 month, there were reductions in psychotic symptoms, depression, and general psychopathology.
Video-based mobile health interventions for people with schizophrenia: bringing the “pocket therapist” to life	Ben-Zeev et al. (2018)	A parallel convergent mixed-methods design using mHealth	*N* = 10 Dx = SSD	To investigate if video-based mHealth interventions are feasible, acceptable, and engaging to people with SZ.	Participants were provided the FOCUS–AV app that had a choice in intervention modality—either videos showing clinicians who “speak” directly to participants or written content. Participants used the intervention in their own environments and were provided feedback on their experiences.	Three SQ	Video-based mHealth may be a feasible, usable, acceptable, and engaging method to implement flexible interventions to people with SZ. Videos were found to be more personal, engaging, and helpful for illness management. Video and written modalities were rated as equally easy to use, understand, and motivating. Participants preferred video interventions to written mHealth interventions in most aspects. All participants used the on-demand intervention videos regularly (66.7% of all self-initiated use).
Mobile application for self-management in schizophrenia: a pilot study	de Almeida et al. (2018)	Quantitative pre-experimental using mHealth	*N* = 9 Dx = SZ	To present the feasibility and efficacy of weCOPE.	Participants were taught how to use weCOPE and used the application under the supervision of their case manager. At the end of the experiment, the final assessments were conducted by the same evaluators in the same environment to control for outlying factors.	Recovery Assessment Scale, GES, SSS, PSS, and PANSS	The SSS had significant differences in the subscales for ‘intimacy’ and “satisfaction with family” except for the subscale “social activities.” Improvements in perception of recovery, self-esteem, social support, empowerment, and performance. For the Recovery Assessment Scale there was increases in the subscales “personal confidence and hope,” “goals and success orientation,” and “nondomination by symptoms,”.
Design of a virtual reality system for affect analysis in facial expressions (VR-SAAFE); application to schizophrenia	Bekele et al. (2017)	Usability and pilot testing using nVR	*N* = 24 Dx = SZ or SSD	To design and develop a SZ intervention system for emotion recognition tasks, and to assess the usefulness could be helpful in understanding the underlying emotion processing mechanism of patients with SZ.	The VR-based system had a total of 20 trials corresponding to five emotional expressions. In each trial, the character first narrated a story that was related to the expression. The avatar had a neutral emotional face during story telling. Subjects were asked what emotion they thought the avatar showed and to rate how sure they were in their choice. Another session included just the facial expression without the story.	Three performance metrics: identifying emotional expressions correctly, their confidence with this and the amount of time, eye gaze, heart rate and skin conductance.	Participant and controls demonstrated no significant differences in confidence ratings for emotional recognition. The control group was significantly more confident than the participants in identifying the correct emotion when there was no story. VR stimuli were successful in eliciting emotional responses compared with baseline in participants.
Development and usability testing of focus: a smartphone system for self-management of schizophrenia	Ben-Zeev et al. (2013)	Developmental and usability testing using mHealth	*N* = 12 Dx = SZ or SSD	To create an mHealth intervention, FOCUS that would be suitable and usable for individuals with serious mental illness.	There were three stages described: stage 1 was a needs assessment, Stage 2 was developing FOCUS, and stage 3 was usability testing. The participants used FOCUS and provided verbal commentary and usability ratings.	SQ	Individuals with no prior experience with smartphones can learn to use a mobile illness self-management system quickly. Larger buttons and more visual aids were reported to help with using the app. Participants saw value in the FOCUS system and were confident they could use it for illness self-management of illness, and were interested in doing so in the future.
The efficacy of SMS text messages to compensate for the effects of cognitive impairments in schizophrenia	Pijnenborg et al. (2010)	RCT using mHealth	*N* = 62 Dx = SSD	To improve functioning in daily life through SMS text messages and to increase the percentage of goals achieved in daily life, that would then decrease once the intervention stopped. Also if participants who benefit from the SMS text messages would also improve on indirect outcome measures of self-esteem, community functioning, and psychiatric symptoms.	The first session explained the project, and the following sessions included psychoeducation. For each participant, a schedule of SMS text messages was developed and entered into a website. For each goal, two prompts were sent. Three weeks after SMS stopped, the number of goals achieved was scored again over a period of 2 weeks. The final 2 sessions focused on reading SMS for training.	Short version of the Groninger Intelligence Test, Verbal Memory Test, Continuous Performance Test, Six Elements Test, Trail Making Test, Faux Pas Test, Prosody Test, FEEST, Client Motivation for Therapy Scale, SFS, and PANSS	Participants positively rated the prompts. When prompted with SMS participants achieved significantly more of their goals in daily life, although goals were still not fully achieved. Prompting led to a favorable effect on leisure activities but did not lead to a significant increase in medication adherence, attendance at the training program, or the inhibition of undesired behavior.
Internet-based self-help for psychosis: findings from a randomized controlled trial	Westermann et al. (2020)	RCT using computer-based training program	*N* = 50 Dx = SSD	To evaluate an IBI for psychosis in patients with acute positive symptoms and if an iCBTp intervention would be used regularly, reduce the severity of positive symptoms compared with a waiting period, and would have minimal negative experiences.	Intervention group had access to an Internet-based self-help platform and a smartphone app. There were 11 iCBT modules, including an introductory module about CBT principles and another related to social competence. The introductory CBT module had to be completed. A typical module consisted of 21 pages (i.e., webpages) and took about 30 to 60 min to complete.	PANSS-PF, LSHS, PC, RSE, ISI, PSWQ-A, PHQ Secondary outcomes: MAAS, ICQ, K-INK, Psychol. QoL, and ISMI	A decrease in self-reported auditory hallucinations and paranoia with small to medium effect sizes was found. Access immediately or delayed to the intervention did not have a significant effect on any of the outcomes. Adherence was mixed. A significant effect on a subset of secondary outcomes that measure psychological resources (mindfulness, self-esteem, SS) was found in favor of iCBTp.
Mobile assessment and treatment for schizophrenia (MATS): a pilot trial of an interactive text-messaging intervention for medication adherence, socialization, and auditory hallucinations	Granholm et al. (2012)	Pilot quantitative study using mHealth	*N* = 55 Dx = SZ or SSD	To evaluate changes in medication adherence, socialization and auditory hallucinations after using the MATS intervention for 12 weeks.	Twelve text messages were sent Monday through Saturday, with each message targeting one of the three possible interventions relating to medication adherence, socialization, or auditory hallucinations. All three interventions were delivered in random order in the morning, afternoon, and evening. Participants received up to total of 840 text messages. If the person did not respond to the first two questions, no more were sent that day.	Daily Ambulatory Monitoring Outcome Assessment Questionnaire, PANSS, BDI-2, ILSS and ANART	Low-intensity text-messaging interventions led to significant improvements for medication adherence, socialization, and auditory hallucinations. Significantly increased having 4 or more social interactions. MATS was associated with improvement in socialization outside of the home and the probability of having only 1 social interaction per day decreased over 10%. Also decreased reporting that socializing is 'a waste of time' or 'dangerous'.
Efficacy of prime, a mobile app intervention designed to improve motivation in young people with schizophrenia	Schlosser et al. (2018)	RCT using mHealth	*N* = 38 Dx = SSD	To test the efficacy of the mobile intervention called PRIME and the feasibility of conducting a completely remote clinical trial for individuals with SZ.	Participants logged onto PRIME and created an account. Participants had to log on once a week for 12 weeks and engage in the application or the online web forum.	Trust Task, MAP-SR, RFS; QOL-A, BDI-2, R-SES, and PANSS	Increased effort of future social interactions, increased anticipatory pleasure from positive outcomes and increased learning. PRIME was used about 4 days a week, over 5,000 messages were sent to coaches and approximately 500 to peers. Participants appreciated interacting with other young people with SZ and to have on-demand coaching from clinicians. Significant improvements in depression, defeatist beliefs, self-efficacy, and motivation.
Development of the motivation and skills support (MASS) social goal attainment smartphone app for (and with) people with schizophrenia	Fulford et al. (2020)	Usability and pilot testing using mHealth	*N* = 8 Dx = SZ or SSD	To create a smartphone app, called MASS, that could aid in increasing SS and motivation using evidence-based approaches and stakeholder input to improve social functioning in SZ.	Stage 1 collected stakeholder input from clinicians and individuals with SZ. Stage 2 was creating the application and stage 3 was pilot testing the application. Participants selected 1 of the 11 available social goals that were meaningful. After the 2 weeks, participants provided feedback to help with revisions of the application.	Focus groups, individual interviews, and EMA	Rated SST in a video format and prompts of the steps to complete goals as helpful. Suggestions for improving the app included adding more video content, less daily notifications and providing more guidance on social goals. Demonstrated acceptability, engagement, and high satisfaction. Reminding participants of their past social experiences were rated as very helpful.

*Note.* Dx = Diagnosis; SSD = schizophrenia spectrum disorders; iVR = immersive Virtual Reality; VE = virtual environment; SZ = Schizophrenia; SST = social skills training; PANSS = Positive and Negative Symptoms Scale; SQ = Subjective Questionnaire; VR = virtual reality; nVR = non-immersive Virtual Reality; AI = Assertion Inventory; SSIT = Simulated Social Interaction Test; SADS = Social Avoidance and Distress Scale; SFS = Social Functioning Scale; SS = social skills; RCT = randomized controlled trial; SST-VR = Social Skills Training Virtual Reality Role-playing; SST-TR = Social Skills Training Traditional Role-playing; SBS = Social Behavior Scales; RAS = Rathus Assertiveness Schedule; SPSI-R = Social Problem Solving Inventory-Revised; BIS/BAS = Behavioral Inhibition Scale/Behavioral Activation Scale; TEPS = Temporal Experience of Pleasure Scale; GAMES = Gaming Attitudes, Motivations, and Experiences Scales; INT = Integrated Neurocognitive Therapy; TAU = Treatment As Usual; PFA = Picture of Facial Affect Test; Emorec = Emotion Recognition Questionnaire; SCST-R = Schema Component Sequencing Task-Revised; AIHQ = Ambiguous Intentions Hostility Questionnaire; CR = Cognitive Remediation; BAC-J = Brief Assessment of Cognition in Schizophrenia; LASMI = Life Assessment Scale for Mentally Ill; MEMS = Mobile Enhancement of Motivation in Schizophrenia; GCT = Collaborative Goal Technology; QLS = Quality of Life Scale; MAP-SR = self-report Motivation and Pleasure Scale; ILFE = I am Learning Facial Expressions; SANS = Scale for Assessment of Negative Symptoms; SAPS = Scale for Assessment of Positive Symptoms; BPRS = Brief Psychiatric Rating Scale; SDLT = Serial Digit Learning Test; WCST = Wisconsin Card Sorting Test; CAPE = Community Assessment of Psychic Experiences; WAIS = Wechsler Adult Intelligence Scale; ESM = Experience Sampling Method; SMARTapp = Schizophrenia Mobile Assessment and RealTime feedback application; N/A = non-applicable; VR SST = Virtual reality social skills training; TR-SST = Traditional Social Skills Training; DPAS = Defeatist Performance Attitude Scale; ABS = Asocial Beliefs Scale; CAINS-MAP = Clinical Assessment Interview for Negative Symptoms Motivation and Pleasure; CDS = Calgary Depression Scale for Schizophrenia; A-QLS = Abbreviated Quality of Life Scale; BDI-2 = Beck Depression Inventory–Second Edition; ISI = Insomnia Severity Index; BMQ = Brief Medication Questionnaire; FOCUS-AV = FOCUS-Audio/Video; ES = Empowerment Scale; GES = General Self-Efficacy Scale; SSS = Social Support Satisfaction Scale; PSS = Personal and Social Performance Scale; VR-SAAFE = Virtual Reality System for Affect Analysis in Facial Expressions; SMS = short message service; FEEST = Facial Expression of Emotion: Stimuli and Test; IBI = Internet-based Intervention; iCBTp = Internet-based Cognitive-behavioral therapy for psychosis; PANSS-PF = positive factor of the PANSS; LSHS = Launay-Slade Hallucination Scale; PC = Paranoia Checklist; RSE = Rosenberg Self-Esteem Scale; ISI = Insomnia Severity Index; PSWQ-A = Penn State Worry Questionnaire abbreviated; PHQ = Patient Health Questionnaire; MAAS = Mindful Attention and Awareness Scale; ICQ = Brief Interpersonal Competence Questionnaire; K-INK = Brief version of the Incongruence Questionnaire; Psychol. QoL = Psychological Quality of Life; ISMI = internalized stigma of mental illness; MATS = Mobile Assessment and Treatment for Schizophrenia; ILSS = Independent Living Skills Survey; ANART = American national Adult Reading Test; MAP-SR = Motivation and Pleasure-Self Report scale; RFS = Role Functioning Scale; QOL-A = Quality of Life Scale-Abbreviated; R-SES = Revised Self-Efficacy Scale; MASS = Motivation and Skills Support; EMA = ecological momentary assessment.

### Educational Technology

Four types of ETs were identified that could teach SS to individuals with SZ. The first type were computer-based training programs, which included completing cognitive exercises on a computer with cognitive behavioral therapy principles embedded to increase SS (Iwata et al., 2017; Mueller et al., 2015; Nahum et al., 2014; Prikken et al., 2019; Westermann et al., 2020). In addition, web-based games involve playing online games that use principles of errorless learning, positive reinforcement, and self-instruction. The games were designed to address SZ social cognitive challenges (Choi et al., 2020; Gülkesen et al., 2017).

The second ET was VR, including immersive or non-immersive types. Immersive VR (iVR) is a computer-simulated experience involving a three-dimensional virtual environment (VE), head-mounted display (HMD) and joystick to simulate social interactions to develop and teach SS (Bisso et al., 2020). Non-immersive VR (nVR) includes a monitor that displays a non-3D VE but still allows participants to practice social interactions with virtual avatars (Bisso et al., 2020). mHealth applications were referred to as the use of smartphone communication technologies to promote health through evidence-supported interventions (de Almeida et al., 2018). mHealth applications incorporate written and video elements to share information individuals could access on smartphones. Participants could choose personalized goals and contact therapists for social support (Table 2).

**Table 2.** Educational Technologies.

Title	Name	Educational technology type	Educational technology type/characteristics
The efficacy of computerized cognitive drill and practice training for patients with a schizophrenia-spectrum disorder: A meta-analysis (Prikken et al., 2019)	N/A	Computer-based training program	A remediation-based approach where people complete repeated tasks on a computer that could focus on cognitive domains such as working memory and attention.
VR-based conversation training program for patients with schizophrenia: a preliminary clinical trial (Ku et al., 2007)	N/A	iVR	A VE-based conversation training program that shows people a screen where they can select conversation skills to “mimic”' conversation scenarios seen on the screen with avatars rendered on a PC. People engage in the task on a big screen using a joystick and HMD to command or control the avatar using the “talk” button to initiate talking with others in the VE.
A virtual reality-integrated program for improving social skills in patients with schizophrenia: a pilot study (Rus-Calafell et al., 2014)	N/A	nVR	The VR-based program involves using a laptop with a monitor, 3D glasses, and headphones. This VR system allows people to practice social interactions with virtual avatars, encourages progressive learning of the social skills and provides positive or negative reinforcement.
A virtual reality application in role-plays of social skills training for schizophrenia: a randomized, controlled trial (Park et al., 2011)	N/A	iVR	This VR-based program utilizes a computer, joystick, wearable HMD for displaying the VE with the purpose to make it a more immersive manner, and a wearable position tracker for following the head direction in real time. SST-VR was different from SST-TR in that it included core features of role-playing games. For example, the participant wearing the HMD was provided with a joystick and buttons to operate their avatar, which produced the first-person perspective view. By using the joystick and buttons, the individual freely moved and interacted with avatars in the virtual space.
A novel, online social cognitive training program for young adults with schizophrenia: a pilot study (Nahum et al., 2014)	SocialVille	Computer-based training program	A laptop and social cognitive training software that was designed to treat social cognition using 19 computerized exercises to improve the speed and accuracy for cognitive functions relating to the processing of social information. The SocialVille exercises target social cognitive domains of affect perception including social cue perception. Each SocialVille session is composed of six exercise blocks, each taking about 10 min to complete.
Habits and attitudes of video gaming and information technology use in people with schizophrenia: cross-sectional survey (Choi et al., 2020)	N/A	Web-based game	The internet is used for videos and web-based gaming. The most favorable gaming platforms are cellular phones, followed by computer, arcade cabinets, and Facebook. The most favorable game genre are action games.
One-year randomized controlled trial and follow-up of integrated neurocognitive therapy for schizophrenia outpatients (Mueller et al., 2015)	CogPack	Computer-based training program	A computer-based exercise program that utilizes INT, a computerized neuro-cognitive exercises to improve neurological and social-cognitive domains through drill-and-practice approaches. It targets the social-cognitive domains of emotion processing, social perception, theory of mind, social attributions, and social schema. These domains were divided into four therapy modules that increased in complexity. It begins with an introductory session to enhance people’s understanding of the importance of the domain in everyday life and to increase insight into their own cognitive abilities. The consecutive sessions consists of people learning individual coping strategies to compensate for any challenges in cognition and apply these strategies in interactive group exercises to increase the automaticity of the strategies. Each exercise is partially computer-based using the CogPack program. Homework in the form of in vivo exercises was used to help transfer of the strategies to everyday life.
Efficacy of cognitive rehabilitation using computer software with individuals living with schizophrenia: a randomized controlled trial in Japan (Iwata et al., 2017)	CogPack	Computer-based training program	The software program, CogPack (Japanese version), provided the CR training through a computer. This CR training was based on the Thinking Skills for Work program, which was developed for rehabilitation of higher brain dysfunction, and it includes 64 cognitive tasks to improve memory, attention, psychomotor speed, executive and social functioning.
Mobile enhancement of motivation in schizophrenia: a pilot randomized controlled trial of a personalized text message intervention for motivation deficits (Luther et al., 2020)	MEMS	mHealth	An application that uses web-based text messaging services to provide reinforcement and cues people to try to complete individualized pre-determined goals. Daily text-messages are sent to remind people about the sub-goals they set to complete that day, ask about the level of effort the goal would take to complete (on a scale of 1–10), and then send positive encouragement. The text-messages also ask when users want to complete their goal that day, how they can assess when they reach their subgoal, and how much effort users put into achieving their goal. If people do not complete a subgoal then they are asked what might help them reach their subgoal and if the subgoal could be broken down further. When people do complete a goal, positive reinforcement messages were texted to reinforce and support their achievement.
A web-based game for teaching facial expressions to schizophrenic patients (Gulkesen et al., 2017)	ILFE	Web-based game	Utilizes an educational software website that is designed for teaching and improving facial expressions skills. It is composed of eight computer games (name this expressing, find the correct expression, carry the correct image, match the image and the expression, find the same expression, find the different expression, balloons, and memory cards) to play on a computer. The games were designed using principles of errorless learning, repetition, feature abstraction, direct positive reinforcement, and self-instruction. The games were designed to address SZ social cognitive challenges.
An ecological momentary intervention incorporating personalized feedback to improve symptoms and social functioning in schizophrenia spectrum disorders (Hanssen et al., 2020)	SMARTapp	mHealth	The SMARTapp is a smartphone application that delivers six ESM questionnaires prompted by a beep and personalized interactive ESM-derived feedback in the form of two tailored prompts. The SMARTapp also includes cloud-based data storage and a reporting module to collect ESM data (thoughts, feelings, and behavior) in everyday life.
Immersive virtual reality applications in schizophrenia spectrum therapy: a systematic review (Bisso et al., 2020)	N/A	iVR	N/A
Mobile-assisted cognitive behavioral therapy for negative symptoms: open single-arm trial with schizophrenia patients (Granholm et al., 2020)	CBT2go and mCBTn	mHealth	mCBTn combines weekly in-person group therapy sessions with the CBT2go smartphone app. The app extends skills learned in therapy such as recovery goal setting, thought challenging, scheduling activities and social interactions, and interventions to target defeatist attitudes and decrease negative symptoms. mCBTn targets defeatist attitudes to improve negative symptoms in schizophrenia. The CBT2go app is used to prompt and track goal-directed activities in the community, help to facilitate homework completion involving community practice and prompt performance and reflection of enjoyable activities and social interactions planned in group. The CBT2go app used personalized statements developed in group to challenge social disinterest and defeatist attitudes in real-world environments in the moment. After groups, therapists could enter personalized comments that participants made in group into a web-based dashboard which is used to challenge low motivation, anticipatory pleasure, or anticipated success of activities.
Feasibility, acceptability, and preliminary efficacy of a smartphone intervention for schizophrenia (Ben-Zeev et al., 2014)	FOCUS	mHealth	FOCUS was designed for people with SZ and offers both prescheduled and on-demand illness management interventions targeting auditory hallucinations, social functioning, medication use, mood problems, and sleep concerns. Based on their self-ratings, FOCUS delivers suggestions and support statements in written text and images. It is composed of three applications that can be installed onto smartphones and web-based dashboards. The first app prompts users to engage in the application daily using auditory and visual notifications on the screen. The second app uses “interactive algorithms” to create short assessments and interventions that people can progress through on the smartphone. The third application is Quick Tips that allows access to illness self-management resources and suggested coping strategies from a variety of options. Users are sent a prompt to encourage engagement of the application that can be ignored. If users engage, the system will launch a brief assessment to ask about their current status. The intervention content is “on-demand” by going to the FOCUS homescreen and selecting any of the treatment target pictures.
Video-based mobile health interventions for people with schizophrenia: bringing the “pocket therapist” to life (Ben-Zeev et al., 2018)	FOCUS-AV	mHealth	FOCUS–AV contains video adaptations for all the FOCUS content and including the daily prompts. After users provide a response, they are given the option to choose between a written intervention or a video version. If they select the written intervention, users navigate at their own pace through a sequence of several screens. If users choose video, then they watch a video that contains the same intervention information as the written format.
Mobile application for self-management in schizophrenia: a pilot study (de Almeida et al., 2018)	weCOPE	mHealth	An illness self-management mobile application. It includes four modules: symptom monitoring, problem-solving, anxiety management, and goal setting and incorporates CBT principles. It also has a feature that allows contact with therapists for crisis situations. It was developed to address the treatment gap and reduce the costs of self- management interventions.
Design of a virtual reality system for affect analysis in facial expressions (VR-SAAFE); application to schizophrenia (Bekele et al., 2017).	VR-SAAFE	nVR	Presentation of realistic facial emotional expressions that can be controlled and embedded in different contexts. It is integrated and synchronized with physiology and eye gaze data collecting abilities. It is a VR-based task presentation platform that can minutely control facial expressions of an avatar with or without accompanying verbal communication, with an eye-tracker to quantitatively measure a participant’s gaze and uses a set of physiological sensors to help indicate a user’s affective states to allow in-depth understanding of the emotion recognition mechanism of people with SZ.
Development and usability testing of focus: a smartphone system for self-management of schizophrenia (Ben-Zeev et al., 2013)	FOCUS	mHealth	A smartphone application designed to support illness self-management among people with SZ. Please refer above for a description of the application.
The efficacy of SMS text messages to compensate for the effects of cognitive impairments in schizophrenia (Pijnenborg et al., 2010)	SMS text messages	mHealth	SMS text messages are short text messages (up to 160 characters), sent to smartphones. The application involves creating an individualized goal and entering it into a website. Then two prompts are sent, the first is sent about an hour before the goal behavior should occur and the second about 10 min before the goal behavior should occur so then people could engage in the necessary activities needed to complete their goal.
Internet-based self-help for psychosis: findings from a randomized controlled trial (Westermann et al., 2020)	iCBTp	Computer-based training program	The IBI called iCBTp is a self-help platform delivered through a computer that has 11 modules including introduction, paranoid ideation, voice hearing, self-esteem, sleep hygiene, metacognition, depression, mindfulness, worrying, social competence, and relapse prevention. Content of each module consists of 21 webpages of texts, pictures, explanatory videos, and interactive worksheets embedded in the module called inline worksheets. The iCBTp also included an optional smartphone app that includes worksheets to facilitate transfer from the program to daily functioning, which were also available via the self-help platform.
Mobile assessment and treatment for schizophrenia (MATS): a pilot trial of an interactive text-messaging intervention for medication adherence, socialization, and auditory hallucinations (Granholm et al., 2012)	MATS	mHealth	An application that incorporates cognitive behavioral techniques to provide prompts through text-messaging to encourage healthy behaviors targeting three treatment goals: increasing medication adherence and socialization and decreasing auditory hallucinations. Users received three sets of interactive text messages that contained personalized evidence to challenge unhelpful beliefs. If the user did not reply to the first or second prompt, the next messages were not sent. If the participant reported positive outcomes (i.e., they socialized with four or more people), users will still receive a second prompt requiring a response asking what interventions are helping and what coping strategies users are using.
Efficacy of prime, a mobile app intervention designed to improve motivation in young people with schizophrenia (Schlosser et al., 2018)	PRIME	mHealth	It is designed to improve motivation and quality of life in the early course of SZ through positive social reinforcement to engage and sustain goal-directed behavior. Users select and document progress on small, self-determined goals relating to health and wellness, social relationships, creativity, and productivity. The application includes features such as video and written content, and a platform for users to message each other and clinicians. Users create a username, upload a profile picture, and select their interests, goals, and symptoms, and can write a short biography. It is based on CBT principles such as behavioral activation, mindfulness, and psychoeducation to help users overcome the daily obstacles that can challenge goal progress and improve health outcomes.
Development of the motivation and skills support (MASS) social goal attainment smartphone app for (and with) people with schizophrenia (Fulford et al., 2020)	MASS	mHealth	Includes 11 social goals that fit into categories related to friends, family, and romantic relationships. The app provides SST video as well as written content of specific social skills to support social goal completion. Also includes reminders or prompts of social goals and information to help with goal planning. Notifications appeared three times per day which directed users to content focused on their identified social goal and steps. Within MASS, a user could select from an array of specific steps related to their overarching social goal (e.g., “make a new friend by attending events you’re interested in”) to attempt or complete before the next notification. Summaries of prior experiences were provided through the app to help support engagement and motivation to work on social goals. When low expectations or motivation is reported on a social goal, the app provides summaries of prior positive affect experiences in the context of social goal progress.

*Notes.* N/A = not applicable; VR = virtual reality; iVR = immersive Virtual Reality; VE = virtual environment; PC = Paranoia Checklist; HMD = head mounted display; nVR = non-immersive Virtual Reality; SST-VR = social skills training virtual reality role-playing; SST-TR = social skills training traditional role-playing; CR = cognitive remediation; MEMS = mobile enhancement of motivation in schizophrenia; ILFE = I am learning facial expressions; SZ = schizophrenia; SMARTapp = Schizophrenia Mobile Assessment and RealTime feedback application; ESM = experience sampling method; CBT = cognitive behavioral therapy; mCBTn = mobile-assisted cognitive behavioral therapy for negative symptoms; FOCUS-AV = FOCUS-audio/video; VR-SAAFE = virtual reality system for affect analysis in facial expressions; SMS = short message service; iCBTp = Internet-based Cognitive-behavioral therapy for psychosis; IBI = Internet-based Intervention; MATS = Mobile Assessment and Treatment for Schizophrenia; PRIME = Personalized Real-Time Intervention for Motivational Enhancement; SST = social skills training; EMA = ecological momentary assessment.

### Themes

There were four key themes presented in the data, including: illness self-management, adjunct therapy approach, personalized goals, and motivation.

#### Illness self-management

Illness self-management skills include increasing social behaviors and engagement and managing SZ symptoms (Ben-Zeev et al., 2014) and was defined as being active in self-monitoring, avoiding high-risk stressors, adhering to medication, and implementing strategies when mental health challenges increase (Ben-Zeev et al., 2013). mHealth applications were identified as valuable tools to support SS (de Almeida et al., 2018; Hanssen et al., 2020; Schlosser et al., 2018). For example, the weCOPE application included four modules on illness self-management and indicated increases in social satisfaction with family, improvements in self-esteem, social support, and empowerment. In addition, statistically significant increases were found in the subscales “personal confidence and hope” and “willingness to ask for help” (de Almeida et al., 2018). Computer-based training programs were another ET used to increase illness-self management strategies. The internet-based intervention, iCBTp, indicated that participants tried to incorporate feedback received through written and video information into their daily lives. As a result, there was a significant effect on SS, mindfulness, and self-esteem (Westermann et al., 2020). The studies indicate when information was provided through an mHealth application or computer-based training program individuals experienced illness self-management in the domain of SS.

#### Adjunct therapy approach

Adjunctive therapy includes technology that is combined with conventional psychosocial treatments to supplement or enhance treatments (Rus-Calafell et al., 2014). Nine studies reported positive results with ETs as an adjunct therapy approach. An RCT by Iwata et al. (2017), identified that cognitive remediation using a computerized cognitive training software (CogPack) combined with traditional rehabilitation interventions resulted in significant improvements in social functioning behaviors compared with rehabilitation alone (Iwata et al., 2017). A meta-analysis by Prikken et al. (2019), identified there is no convincing evidence for the efficacy of adding computerized drill and practice training programs to standard treatment in clinical settings.

Ku et al. (2007), suggested that implementing a VR system to provide scenario-based conversational skills training could complement standard social skills training (SST) or role-playing interventions. Results demonstrated that the VR-based conversation skills training program could be used for conversation skills training (Ku et al., 2007). A pilot study addressed the effectiveness and utility of a VR-integrated conversation skills program as an adjunct technique and revealed high acceptance of the SST with the VR-integrated program, significant treatment satisfaction, improvements in emotional responses and SS mastery (Bekele et al., 2017; Rus-Calafell et al., 2014).

Results from a systematic review indicated that current evidence cannot yet confirm whether VR treatments are superior to standard or traditional psychosocial interventions for SST. However, emerging evidence suggests that outcomes for VR-based SST interventions were similar if not slightly more effective in improving conversational skills compared with standard interventions (Bisso et al., 2020). Overall, results highlighted that mHealth interventions and VR-integrated programs could be considered adjunctive to conventional SST approaches (Rus-Calafell et al., 2014; Schlosser et al., 2018).

#### Personalized goals

The mHealth applications and computer-based training programs were rated as more feasible, acceptable, and effective if goals could be personalized. mHealth applications ranged from participants independently creating their own goal such as in the PRIME application, to identifying goals from existing goal categories such as with the FOCUS and CBT2go applications. Participants could also choose goals specifically relating to improving SS such as with Mobile Enhancement of Motivation in Schizophrenia (MEMS) and short message service (SMS) text messaging applications and the SMARTapp (Hanssen et al., 2020; Luther et al., 2020; Pijnenborg et al., 2010).

The results of the studies indicated increased effort was put forth by participants to engage in more social interactions per day, resulting in significant improvements in attainment of their personalized social functioning goals (Granholm et al., 2012; Hanssen et al., 2020; Luther et al., 2020). Studies that utilized SMS text messaging and the computer-based training program iCBTp, did not personalize the information or set individualized goals for each participant and the authors stated that personalizing ETs and goals should be incorporated into their technology to increase motivation and engagement to achieve a higher percentage of goals through SS interventions (Pijnenborg et al., 2010; Westermann et al., 2020). Overall, various ETs that allowed goals to be chosen by the participant were associated with achieving a higher percentage of goals related to SS.

#### Motivation

Motivation components within treatment programs for SZ were common throughout this scoping review. Prikken et al. (2019) identified the incorporation of interactive gaming features through a computer-based training program could help overcome amotivation among SZ as these elements were motivating and meaningful. Authors suggested that iVR and web-based gaming motivational features of being present in the simulated VE or gaming software could elicit emotional and motivational stimuli and foster the development of interpersonal skills in real-world situations (Choi et al., 2020; Ku et al., 2007). Participants engaged more in the SST-VR group than the SST traditional role-play (SST-TR) group, which suggested that VR role-plays could increase motivation in SST (Bisso et al., 2020; Park et al., 2011).

Nahum et al. (2014), tested the feasibility and efficacy of a novel, online social cognitive training program called SocialVille. This pilot study identified that individuals with SZ showed improvements in social functioning and motivation. Mueller et al. (2015), evaluated the efficacy of a manualized cognitive remediation group therapy program called CogPack. The study resulted in high acceptability due to an emphasis on the development and maintenance of intrinsic motivation by considering individuals’ daily experiences and fostering group cohesion. Participants were provided a FOCUS-Audio/Video application that had a choice in either selecting video or written content modalities to deliver the intervention. The study identified that video and written modalities were equally rated as motivating and easy to understand by individuals with SZ.

Smartphone applications also enhanced participation and motivation for SZ treatment programs when participants received support through SMS. The mHealth applications incorporated SMS features with the goal of initiating and increasing social interactions with others. Using SMS as part of the intervention resulted in increased therapeutic rapport, motivation, and a sense of belonging which could help initiate social interactions for individuals with SZ (de Almeida et al., 2018; Luther et al., 2020). Fulford et al. (2020), incorporated text messaging, videos, and evidence-based approaches such as SST and cognitive behavioral therapy for psychosis into MASS. Results demonstrated that a key feature of MASS was SMS that reminded participants of their past social pleasures to increase motivation. Overall, smartphone application interventions have the potential to improve social functioning through increasing social motivation in individuals living with SZ (Fulford et al., 2020).

## Discussion

The purpose of this scoping review was to provide an overview of the extent, range, and nature of evidence of existing ETs in the context of social skills (SS) interventions for individuals with SZ. Results suggested that people with SZ responded favorably to using mHealth applications to increase contact with services and illness self-management (Firth et al., 2016). Targeting SS and social competence of individuals with SZ can help compensate for the effects of SZ symptoms and help improve social affiliation, interpersonal supports and QoL (Kopelowicz et al., 2006). These findings correlate with the belief that illness self-management programs should aim to improve skills that help to pursue personal goals and not only focus on reducing symptoms (Mueser et al., 2002). In mental health practice, occupational therapists use the recovery model, which includes principles of hope, client-centered and holistic care. Recovery principles can be applied by occupational therapists for illness self-management approaches when using ETs by incorporating client-centered therapeutic modalities such as personalized health applications in mental health practice (Champagne & Gray, 2016). Illness self-management using mHealth applications and computer-based training programs can help persons with SZ develop better social and professional relationships and emerge as potentially new intervention approaches in the treatment of SZ (Mueser et al., 2002).

The existing ETs emerged as an adjunctive therapy component to conventional SS interventions for SZ. Adjunctive therapy includes components that are combined to supplement, enhance, or complement traditional psychosocial treatments (Granholm et al., 2020; Iwata et al., 2017). In this review, ETs were associated with greater improvements in social functioning when used as an adjunct to therapy compared with traditional SST approaches alone (Granholm et al., 2020; Iwata et al., 2017; Schlosser et al., 2018). ETs, particularly mHealth applications, have the potential to strengthen and shorten traditional, intensive approaches and improve access to evidence-based psychosocial interventions for SZ (Ben-Zeev et al., 2014; Granholm et al., 2020). Although the mHealth applications were used as an adjunct to in-person services, these ETs could be used as stand-alone community-based treatments (Ben-Zeev et al., 2014). Smartphone technology could improve SS in individuals with SZ, through extending the access of services and providing adjunctive support to existing, psychosocial treatment programs for SZ (Firth et al., 2016). When undergoing adjunctive therapy, occupational therapists can contribute to the development of a decision-making framework to choose suitable ETs for individuals with SZ (Chivilgina et al., 2021).

The mHealth applications and computer-based training programs resulted in higher rates of acceptability and usability if goals could be personalized. Researchers suggested that incorporating this feature was the next step to increasing the effectiveness of ETs (Westermann et al., 2020). Creating ETs to teach SS that allow participants to set goals is related to a person-centered care approach, which assumes clients are capable and knowledgeable in their own care and is a key component of OT practice (Mroz et al., 2015). This is consistent with literature that clients have preferences over personalized treatment goals (Bridges et al., 2013).

The importance of considering motivation within treatment programs is an important component ETs aim to address. Amotivation is a factor affecting QoL, social behaviors and engagement for individuals with SZ; however, research suggested that it is not commonly considered when developing treatment programs (Prikken et al., 2019). The VR, computer-based training program, and mHealth application emphasized fostering intrinsic motivation for SS training through addressing individual experiences and SS deficits (Prikken et al., 2019). In contrast to psychosocial interventions, findings highlight how ETs provide a method for generalization and continuation of SS for real-time self-management within dynamic social interactions. mHealth applications provide promise in addressing psychosocial outcomes in an on-demand and personalized format that targets social motivation (Fulford et al., 2021).

The use of ETs to teach SS is an emerging area of practice that has the potential to offer a new method of service delivery, pending on future research conducted to support ETs as an evidence-based intervention (Torous & Keshavan, 2016). Majority of the studies (*n* = 14) included were pilot studies, which indicates that the research in this area is new and represents the first step toward validating the treatment approach (Nahum et al., 2014). Nine of the studies reported positive, preliminary results for this area of intervention for SZ or schizophrenia spectrum disorder (SSD), which could warrant future RCTs to establish the efficacy of the ET interventions and extend these findings (Luther et al., 2020). The ETs approach to SZ treatment is emerging and only possible because of the recent and ongoing technical advancement of virtual and smartphone technologies (Ben Zeev et al., 2018). A recent study highlighted the feasibility of remotely delivering treatments to people with SSD which demonstrates that emerging research in ETs is being published (Dabit et al., 2021). Smartphone technology has the potential to significantly improve SS among SZ, through extending the reach of services and providing adjunctive support to evidence-based psychosocial interventions (Firth et al., 2016). Due to limited studies in this area of research, the results should be regarded with caution until more rigorous studies have been conducted to confirm the emerging results obtained in this scoping review (Torous & Keshavan, 2016). The findings suggested that with further development and validation, ETs could facilitate more naturalistic SS interventions for people with SZ than clinical psychosocial services at a fraction of the cost (Ben-Zeev et al., 2014). Occupational therapists could have a role in the development of therapeutic applications because of their knowledge in various occupational demands with diverse populations (Seifert et al., 2017). As advancements with ETs emerge, this could expand the range of therapeutic options for occupational therapists to provide SST to people with SZ (Ben-Zeev et al., 2014).

This scoping review presented limitations that should be noted. First, the databases used for the search were CINAHL Plus and Ovid MEDLINE; however, the inclusion of other databases may have identified other articles. In addition, the results are dependent on a list of search terms and different search terms may have led to different results. Finally, this scoping review extracted data from published literature up until December 2020, and new evidence may have been published since this analysis to augment or change these findings.

## Implications for OT

Occupational therapists often assist individuals with SZ in developing and maintaining life skills and SS. Occupational therapists may have little experience with diverse ETs, resulting from limited exposure in OT academic training (Glegg et al., 2013). The implications of the scoping review for the field of OT are that ETs could be used as a potential intervention approach to target SS development among individuals with SZ (Fulford et al., 2020). As ETs continue to develop, they offer novel opportunities that will enable occupational therapists to provide continuous assessment and treatment (Ben-Zeev et al., 2014). Mental health care providers including occupational therapists could identify potential ETs that can be utilized through their practices (Ben-Zeev, 2018).

## Conclusion

There is not one ET approach that is superior to the other types or other psychosocial interventions at this time; however, evidence supports that mHealth applications are currently the most researched at this time. The analysis of studies determined that the existing ETs could teach SS to individuals with SZ, and these positive, preliminary results serve as an area of untapped potential for mental health care.

## Supplemental Material

sj-docx-1-otj-10.1177_15394492221108389 – Supplemental material for Educational Technologies for Teaching Social Skills to Individuals With Schizophrenia: Scoping Review Click here for additional data file. Supplemental material, sj-docx-1-otj-10.1177_15394492221108389 for Educational Technologies for Teaching Social Skills to Individuals With Schizophrenia: Scoping Review by Nicole Surdyka, Amy Clark and Andrea Duncan in OTJR: Occupation, Participation and Health
