# Supplementary material for: Educational Technologies for Teaching Social Skills to Individuals With Schizophrenia: Scoping Review
Source: OTJR (Thorofare N J). 2022 Jul 26;43(1):127–43. doi: 10.1177/15394492221108389 (PMC9729979; doi:10.1177/15394492221108389)
Supplement: sj-docx-1-otj-10.1177_15394492221108389 – Supplemental material for Educational Technologies for Teaching Social Skills to Individuals With Schizophrenia: Scoping Review [file sj-docx-1-otj-10.1177_15394492221108389.docx]

Appendix A

*Search Terms*

((schizophrenia) AND (technolog* OR device* OR "computer based technolog*" OR "mobile application*" OR "virtual learning" OR "social media" OR "multimedia technolog*") AND ("social skill*" OR "social interaction*" OR "social skills training" OR "social support*" OR "social behaviour*")).

Appendix B

*Inclusion Criteria*

Studies were included if they were peer reviewed, described the characteristics of the ETs, and stated methodology for intervention administration. Participants were adults ages 18-64 diagnosed with SZ or a SSD. Included studies also had to deliver the interventions virtually and be published after 2005, as technology before 2005 was limited and underdeveloped (Ben-Zeev, 2017).

*Exclusion Criteria*

Studies were excluded if they did not report on the effectiveness of ETs or its methods of intervention, not in English, did not address SS and were administered to populations other than SZ and SSD. Studies were also excluded if they were an abstract only, if there was no full text, no report of outcomes, not a scientific study, a review, and published before the year of 2005.
